# Development of a Patient-Centred Care Plan for Patients Requiring Maintenance Electroconvulsive Therapy Long-Term

**DOI:** 10.1192/j.eurpsy.2024.510

**Published:** 2024-08-27

**Authors:** X. Y. Seet, X. E. Lee, H. Rahman

**Affiliations:** ^1^Department of Psychiatry; ^2^Tan Tock Seng Hospital, Singapore, Singapore

## Abstract

**Introduction:**

Maintenance electroconvulsive therapy (ECT) can be effective and necessary in the long-term for patients with severe and recurrent mood or psychotic disorders that are not amenable to any other forms of treatment. Patients with such treatment resistance affecting their ability to maintain minimal daily activities may eventually fall within the palliative psychiatric care domain in which advanced medical directives become an important beacon to direct care. There are Psychiatric Advance Directives which allow people with severe mental health conditions to consent to or refuse to consent to hospital admission and psychiatric treatment in the event they lose decision-making capacity and this can be especially important for a potentially controversial treatment such as ECT. However, the focus tends to be on enforcing involuntary treatment and less about a comprehensive long-term care plan. To our knowledge, there is no available framework to structure maintenance ECT as a patient-centred care plan.

**Objectives:**

Our aim is to share the process of development of a patient-centred care plan for patients requiring maintenance ECT. Our objectives are:

1. Constant engagement with patients and family or caregivers
2. Regular reviews of clinical and consent aspects of treatment
3. Advocating for the welfare of patients and respect of values
4. Focus on dignity especially for patients who require treatment well into old age
5. Being prepared for termination of treatment if necessary

**Methods:**

We reviewed our management of previous and existing patients on maintenance ECT and incorporated diligent consent-taking practices. Adopting good practices from known palliative approaches and involving the patient voice helped to form a framework for a patient-centred care plan.

**Results:**

Our patient-centred care plan features half-yearly discussions about the risks and benefits of treatment, as well as an assessment of the patient’s cognition and ability to consent which may change over time. Opportunities for them to share their values and expectations of care and engagement with their caregivers about their quality of life guide the continued treatment. A framework for discussing the disruption or eventual termination of ECT prepares for scenarios where older-aged patients may develop frailty or present with acute, prolonged or devastating medical concerns. This end-of-life care approach manages anticipated psychiatric-specific behavioural concerns and prepares for the possibility of death following the planned termination of ECT for patients who required long-term treatment throughout their life. Lastly, issues of grief amongst caregivers and ethical concerns from medical staff are addressed.

**Conclusions:**

We hope that our patient-centred care plan provides a well-considered conversation and structure for the initiation, continuation and termination of maintenance ECT in the long-term.

**Disclosure of Interest:**

None Declared

